# Investigating the potential of a novel internet-based cognitive behavioural intervention for Dari and Farsi speaking refugee youth: A feasibility study

**DOI:** 10.1016/j.invent.2022.100533

**Published:** 2022-04-01

**Authors:** Tomas Lindegaard, Elisabet Wasteson, Youstina Demetry, Gerhard Andersson, Derek Richards, Shervin Shahnavaz

**Affiliations:** a Department of Behavioural Sciences and Learning, Linköping University, Linköping, Sweden; b Department of Psychology and Social Work, Mid Sweden University, Östersund, Sweden; c Centre for Psychiatry Research, Department of Clinical Neuroscience, Karolinska Institutet, Sweden; d Stockholm Health Care Services, Region Stockholm, The Centre for Psychotherapy, Education & Research, Sweden; e Department of Biomedical and Clinical Sciences, Linköping University, Linköping, Sweden; f E-mental Health Group, School of Psychology, University of Dublin, Trinity College Dublin, Dublin, Ireland; g Clinical Research & Innovation, SilverCloud Health, Dublin, Ireland

**Keywords:** Culturally adapted psychotherapy, Internet-based treatment, Adolescents, Feasibility, Acceptability

## Abstract

**Objective:**

Over half of the world's forcibly displaced persons are under the age of 25, with many suffering from symptoms of psychological disorders. Many refugees from Afghanistan or Iran speak either Dari or Farsi, which are mutually intelligible dialects of the Persian language. Previous research on adult refugees and immigrants have shown that internet-based cognitive behavioural therapy (ICBT) can be a valuable complement to other forms of treatment. However, there is a lack of knowledge if ICBT is a feasible and acceptable treatment for adolescents and young adults with a refugee background.

**Method:**

Fifteen Dari/Farsi-speaking adolescents and young adults between 15 and 26 years of age participated in a feasibility study of a novel individually tailored guided ICBT intervention targeting symptoms of common mental disorders such as anxiety and depression. Self-reported symptoms of anxiety and depression were assessed using the Hopkins Symptom Checklist-25 (HSCL-25) as the primary outcome measure. Four of the treatment participants and three additional non-treatment participants consented to be interviewed regarding the programme's acceptability. The interviews were analysed using Thematic Analysis.

**Results:**

The intervention suffered from low adherence, with only 3 participants completing the post-treatment assessment and with participants completing 0.9 modules on average, which meant that the intended quantitative analysis of the pre to post change was not possible. The thematic analysis resulted in two overarching categories, barriers and facilitators, that each contained four themes and related subthemes. Overall, the intervention was deemed culturally relevant and easy to understand. The most salient barriers to participation across interviews concerned interference of symptoms such as concentration difficulties, low energy, and a lack of human contact and support.

**Conclusion:**

The current version of the ICBT program demonstrated low feasibility and acceptability in the target population, which mainly seemed to be related to the delivery format. Future studies should investigate if a blended treatment format with regular phone/video calls with a therapist can increase adherence to the intervention.

## Highlights

• The feasibility of a novel intervention for Dari/Farsi speaking youth was evaluated.
• The study had low retention and adherence.
• Barriers to adherence included a need for more therapist support.
• The treatment was easy to understand and culturally relevant.
• A blended treatment approach could increase acceptability.

## 1 Introduction

During the last decade, European countries have seen an increased migration and asylum-seeking to several European countries, including Sweden (Statistics Sweden, 2021). Over half of the world's forcibly displaced persons are children or young adults (UNHCR, 2021), with children commonly being defined as persons under 14 and young adults/youth being persons under 25 (United Nations, 2022). Previous research has documented increased post-traumatic stress and depression levels among refugee youth (Bronstein and Montgomery, 2011). Recent research on displaced Syrian children and adolescents revealed a prevalence of 18% suffering from post-traumatic stress disorder (PTSD) and around 70% suffering from an anxiety disorder (Gormez et al., 2018), while a study on Afghan adolescent refugees found that about one third scored above cut-off for a diagnosis of PTSD (Bronstein et al., 2012). Moreover, a recent study found rates of suicide nine times higher among unaccompanied refugee youth, mainly Afghans, in Sweden, compared to the general population (Mittendorfer-Rutz et al., 2020). Risk factors for mental health problems in this group include exposure to violence, death of a loved one, being unaccompanied and experiencing discrimination in the host country (Betancourt et al., 2012; Bronstein et al., 2012; Fazel et al., 2012; Gormez et al., 2018). In addition, the psychosocial stressors associated with the current COVID-19 pandemic can be extra damaging to already vulnerable populations, such as refugee children and youth (Song, 2021).

Despite the mental health challenges facing refugee children and youth, mental health service utilization among migrant and refugee youth is low compared to Swedish-born counterparts (Gubi et al., 2021). In addition, there is also a considerable lack of information regarding effective psychological interventions for this group. Although cognitive behavioural therapy (CBT) is the most well-documented and researched type of treatment for child and adolescent populations (Christ et al., 2020; James et al., 2013; Oud et al., 2019), there is a lack of studies targeting refugee and immigrant populations in this age group. In a recent meta-analysis of psychosocial interventions for war-traumatized refugees and internally displaced minors, the authors found a combined within-group effect size of standardised mean change (SMC) = 0.78 for symptoms of PTSD and SMC = 0.35 for depression (Nocon et al., 2017). However, the authors noted caution in meaningfully interpreting the results due to high heterogeneity across the studies. More recent studies in a European context include a randomized controlled trial of narrative exposure therapy for children with multiple traumas that included children with a refugee background, which found preliminary support for the efficacy of the intervention (Peltonen & Kangaslampi, 2019). Another uncontrolled pilot study investigated the effect of trauma-focused CBT among unaccompanied refugee minors suffering from PTSD, with promising effects (Unterhitzenberger et al., 2019). Regarding internet-based intervention studies, there are no previous studies investigating internet-based interventions targeted towards immigrant and refugee youth. However, possible advantages of an internet-based approach include increased access to interventions in the native language of the recipient while using fewer therapist resources and the fact that internet-based interventions are easier to access anonymously, which might be preferable for groups with high levels of mental health stigma (Andersson and Titov, 2014; Satinsky et al., 2019). In addition, previous research on ICBT for adolescents and young adults under the age of 25 have shown ICBT to be efficacious in reducing symptoms of depression and anxiety compared to inactive control conditions, although more high-quality research is needed with active control conditions and long-term follow-up (Christ et al., 2020).

A critical aspect in the development and adaptation of interventions is to take the target group's social and cultural context into consideration (Bernal et al., 2009; Chu and Leino, 2017). Previous research indicates that cultural adaptation of treatments can increase treatment effects, both in adult populations (Hall et al., 2016) and among youth populations (Huey and Polo, 2008; Pina et al., 2019). Several different frameworks for cultural adaptation exist (for example, Chu and Leino, 2017; Heim and Kohrt, 2019; Resnicow et al., 2000). Nevertheless, there is no scientific consensus on what aspects of treatment are essential to culturally adapt (Heim and Kohrt, 2019). A recent review of cultural adaptation of internet-based interventions found no significant associations between the extent of adaptation and the efficacy or adherence to the intervention (Spanhel et al., 2021). However, no definitive conclusions were made due to the small sample and heterogeneity among studies. In the present study, we used the principles of conceptual equivalence, functional equivalence and linguistic equivalence proposed by (Lonner, 1985) and (Helms, 2015) to guide the cultural adaptation process, similarly to Salamanca-Sanabria et al. (2019). Further, to investigate the acceptability of the intervention, we used the Theoretical Framework of Acceptability (Sekhon et al., 2017), which includes seven dimensions of acceptability: Affective Attitude, Burden Ethicality, Intervention Coherence, Opportunity Cost, Perceived Effectiveness and Self-Efficacy. Feasibility was operationalized as the recruitment rates and adherence to the intervention among the participants.

In conclusion, the current study aimed to investigate the potential of a novel ICBT intervention in reaching Dari and Farsi-speaking refugee youth. Since there are no, to our knowledge, previous studies on ICBT for this group, and also given the overall lack of research on interventions targeting Dari and Farsi speaking refugee youth, the aim of the study more specifically was to investigate the feasibility and acceptability of an adapted version of an individually tailored ICBT intervention aimed at Dari/Farsi-speaking youth. Dari and Farsi are two dialects of the same language (Persian), spoken in Afghanistan and Iran, and are mutually intelligible for both Dari and Farsi speakers. The intervention was developed initially for Arabic-speaking adult immigrants and refugees residing in Sweden (Lindegaard et al., 2020) and later adapted to the age-related and cultural characteristics of the target population. The decision to investigate an individually tailored intervention potentially addresses the high comorbidity among refugee youth (Bronstein and Montgomery, 2011; Păsărelu et al., 2017).

## 2 Method

### 2.1 Study design

The study used a mixed-method design to investigate the feasibility and acceptability of a novel ICBT intervention aimed at Dari/Farsi-speaking youth between 15 and 26 years of age. We opted for a more inclusive age span to facilitate recruitment and also since we deemed it likely that the intervention could be of use also for participants older than 24 years of age. The reason for excluding participants younger than 15 years was that interventions targeting children under the age of 15 often require additional caregiver support outside the current intervention's scope. We originally intended to recruit 40 participants; however, due to difficulties recruiting participants, we decided to settle on 15 participants, which we deemed sufficient to answer the main research question of feasibility. The study included both a pre-post evaluation of treatment effects and qualitative interviews with both participants and non-treatment participants to get an in-depth understanding of the intervention from their perspective.

### 2.2 Participants and recruitment

**Fig. 1:**
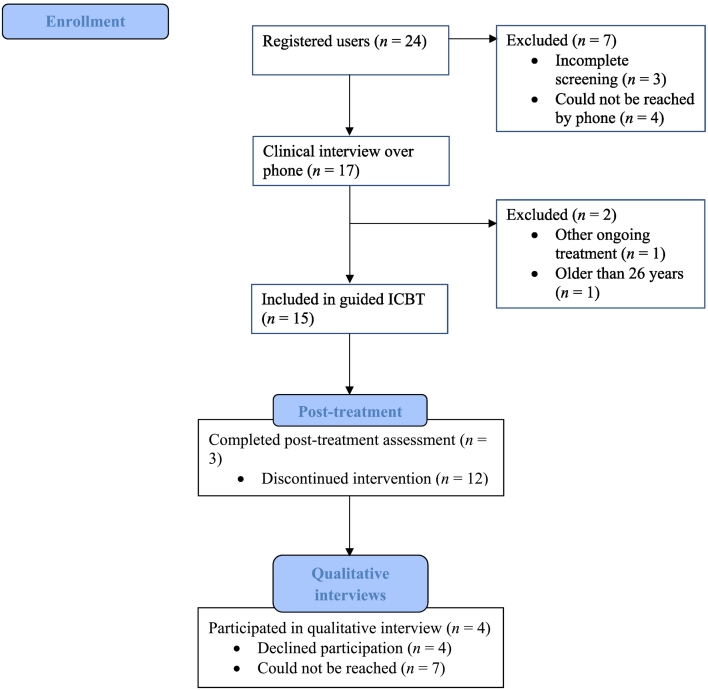
Flow chart of participants throughout the current study.

For a flowchart of participants throughout the study, see Fig. 1. Inclusion criteria for the study included being between 15 and 26 years old, having access to a smartphone, computer, or tablet with an internet connection, having elevated symptoms (>1.55) of depression and/or anxiety on the HSCL-25, speaking Dari or Farsi and residing in Sweden. The cut-off of 1.55 on the HSCL-25 is considered a threshold for a probable psychiatric case (Nettelbladt et al., 1993). Exclusion criteria included severe mental illnesses such as severe depression or psychosis, suicidal ideation, alcohol or substance abuse, and ongoing psychological treatment.

The recruitment period began in February 2021. Information about the study was advertised through social media and to school counsellors and nurses working with immigrant youth and various relevant Non-Governmental Organisations (NGO). Participation was free of charge, and participants did not receive any monetary or other compensation for their participation. The advertisement contained a link to the study website where participants could get more information and sign up for the study. When signing up, participants had to read an informed consent information sheet and consent to participate in the study, which they did online through the study website. They were subsequently sent a link to fill out the pre-treatment assessment on the website. The assessment included several questionnaires, see below, and sociodemographic questions, including questions about ongoing psychological treatment and medication use. Participants deemed to meet inclusion criteria participated in a telephone administered clinical interview lasting between 40 and 60 min before being included in the study. This interview focused on assessing suicidality and screening for severe mental health problems such as severe depression or psychosis. Participants provided information on their motivation and the possibility for them to allocate the time needed to participate in the treatment. All included participants were telephoned by a research team member for a short introduction to the treatment and the treatment format before commencing.

In total, 24 participants registered on the website. Of these, 21 completed the pre-treatment assessment and were scheduled for a telephone-administered clinical interview. However, four participants could not be reached. Of the remaining 17 participants, 15 were deemed eligible and included in the study. Researchers invited all participants to participate in an interview regarding their experiences of participating in the study. In total, four participated.

To better understand the acceptability of the intervention, three further interviews with young adults speaking Dari/Farsi and with similar demographics as the treatment participants contribute to increasing the sample size of interviewees. These three participants were recruited through a Facebook group for Dari and Farsi speaking persons residing in Sweden. One of the study authors posted information in this group that we wanted to recruit Dari and Farsi speaking persons between 15 and 26 years of age to interview them regarding their views of an online intervention targeting mental health problems. For sociodemographic characteristics of participants, see Table 1. For self-reported symptoms at baseline, see Table 2.

**Table 1:** Sociodemographic characteristics of participants.

Baseline characteristics	Included in study (*n* = 15)	Completed qualitative interview (*n* = 4)	Non-treatment participants (*n* = 3)
Age (years): *M* (SD)	21.4 (2.3)	23.3 (2.2)	21.3 (2.5)
Range: *Min-Max*	18–26	21–26	19–24
Gender: *n* (% male)	14 (93%)	4 (100%)	2 (66%)
Highest educational level: *n* (%)			
Elementary school	3 (20%)	2 (50%)	0
Upper secondary school	12 (80%)	2 (50%)	3 (100%)
Vocational education	0	0	0
University (ongoing)	0	0	0
University (completed)	0	0	0
Other	0	0	0
Occupation: *n* (%)			
Student	10 (67%)	3 (75%)	0
Employed	2 (13%)	1 (25%)	1 (33%)
Unemployed	2 (13%)	0	2 (66%)
Sick leave (>3 months)	0	0	0
Parental leave	0	0	0
Other	1 (7%)	0	0
Prior psychological treatment: *n* (% yes)	4 (27%)	0	–
Taking medication: *n*(%)			
Yes	2 (13%)	1 (25%)	–
No, but previously	5 (33%)	1 (25%)	–
No	8 (53%)	2 (50%)	–
Country of origin: *n*(%)			
Afghanistan	14 (93%)	4 (100%)	3 (100%)
Iran	1 (7%)	0	0
Arrived unaccompanied: *n* (%)	14 (93%)	4 (100%)	2 (66%)
Arrived together with family: *n* (%)	1 (7%)	0	1 (33%)
Years lived in Sweden: *M* (SD)	5.33 (1)	5.5 (0.6)	5.7 (0.6)
Residence permit: *n*(%)			
Yes, permanent	3 (20%)	0	2 (66%)
Yes, temporary	4 (27%)	3 (75%)	0
Yes, according to the law on upper secondary education	7 (47%)	1 (25%)	0
No	1 (7%)	0	1 (33%)

**Table 2:** Observed means, standard deviations and Ns for each measure over time.

Measure	Pre-treatment			Post-treatment		
	M	SD	N	M	SD	N
HSCL-25	2.56	0.58	15	2.06	0.39	3
PSYCHLOPS	3.6	1.3	15	2.08	1.63	3
ISI	16.1	7.3	15	16.3	6.43	3
PCL-5	49.5	16.3	10	58	–	1
PG-13	40.6	11.8	5	31	2.83	2
CSQ-3	–	–	–	2.67	0.88	3

Note: HSCL-25 = Hopkins Symptom Checklist-25; ISI = Insomnia Severity Index; PCL-5 = Post-traumatic Stress Disorder Checklist Version 5; PG-13 = Prolonged Grief Disorder-13; CSQ-3 = Client Satisfaction Questionnaire-3.

### 2.3 Treatment and therapists

The treatment was an adapted version of a previously developed ICBT intervention previously researched for adult Arabic-speaking immigrants and refugees in Sweden (Lindegaard et al., 2020). The original intervention consists of 9 modules targeting various problem areas, including depression, anxiety, post-traumatic stress, worry and rumination, stress management, emotion regulation and insomnia, and an introductory and maintenance module (Lindegaard et al., 2020). Each module consists of a brief description of the problem area, a CBT conceptualisation of the factors maintaining the problem, and homework assignments based on standard CBT techniques such as behavioural activation and exposure. Overall, each module contained between 5 and 10 pages of text. Participants were assigned modules based on a combination of their problem presentation and their own stated preference (Păsărelu et al., 2017). The tailoring was done after completing the first module, where the participants could read about the different modules and discuss the appropriate focus with their therapist. The intervention was adapted to fit the target population's needs better for the present study.

Regarding the guidance, all participants had weekly contact through the in-built messaging system in the treatment platform with a therapist who is a licensed clinical psychologist proficient in Dari and Farsi. The therapist's role was to provide encouragement and feedback on the participants' homework assignments. The therapist also sent reminders to participants when they failed to complete the assigned modules in time.

### 2.4 Initial adaptation of the intervention

Before starting the feasibility study, the authors did an initial, top-down adaptation (Salamanca-Sanabria et al., 2019). This adaptation included translating all treatment materials into Dari/Farsi, simplifying the language used to make it more suitable for a youth population, and adding an extra module around prolonged grief and separation anxiety. It was expected that many participants would have recently experienced the death and or separation from an important person (Gormez et al., 2018). This module was based on and inspired by the complicated grief treatment developed by Shear (2010). For additional minor adaptations done before commencing the feasibility study, see Appendix A.

### 2.5 Quantitative measures

All measures were administered at baseline and again at post-treatment, immediately after the end of the 8-week treatment period. Participants were sent up to three reminders via e-mail to complete the post-treatment assessment.

For the primary outcome, the HSCL-25 was used (Mollica et al., 1987). The HSCL-25 consists of 25 items measuring symptoms of anxiety and depression. Items are scored from 1 to 4, and a total score is calculated by adding all scores and dividing by the number of items, with a higher score indicating a higher symptom burden. Cross-cultural studies have shown the HSCL-25 to be reliable and valid among refugee populations (Mollica et al., 1987).

Secondary outcomes included the Post-traumatic Stress Disorder Checklist Version 5 (PCL-5), which measures symptoms of post-traumatic stress according to the DSM-5 (Blevins et al., 2015). It consists of 20 items scored from 0 to 4 with the total score ranging from 0 to 80, with a higher score indicating more symptoms of PTSD. Research has shown PCL-5 to be psychometrically sound with solid reliability and validity in both non-refugee (Blevins et al., 2015) and refugee populations (Ibrahim et al., 2018).

Further, to measure insomnia symptoms, the Insomnia Severity Index (ISI) was used. The ISI consists of 7 items scored from 0 to 4, with the total score ranging from 0 to 28, with a higher score indicating more symptoms of insomnia (Bastien et al., 2001). Research has shown the ISI to be a valid and sensitive measure (Bastien et al., 2001). To our knowledge, the ISI has not been validated in a refugee sample.

In order to measure symptoms of traumatic grief, the Swedish version of Prolonged Grief Disorder-13 (PG-13) was used (Pohlkamp et al., 2018). It consists of 13 items where 11 of these are scored from 1 to 5, and the remaining two are yes/no questions regarding duration and functional impairment. The total score ranges from 11 to 55, with a higher score indicating more prolonged/traumatic grief symptoms. Preliminary investigations in a sample of Swedish parents who had lost a child to cancer have shown the PG-13 to possess good psychometric properties, including a high internal consistency (Pohlkamp et al., 2018).

Finally, the PSYCHLOPS measure asked participants to describe the problem that currently affects them the most (Ashworth et al., 2005). The patient then responds to four questions regarding how affected they are by the problem, duration of the problem, overall wellbeing and how affected they are by a secondary problem, all scored from 0 to 5. An overall score is obtained by adding the sum of the four items and dividing by the number of items with a higher score indicating more problem burden (Ashworth et al., 2005). Preliminary research in a primary care setting indicates that the PSYCHLOPS is a sensitive and reliable measure of change following psychological treatment (Ashworth et al., 2009).

The Client Satisfaction Questionnaire-3 (CSQ-3 Attkisson and Greenfield, 1995) measured overall satisfaction with the intervention. The CSQ-3 is a valid and reliable measure when used in internet-based interventions (Boß et al., 2016) and measures from 1 (low satisfaction) to 4 (high satisfaction); a mean score is calculated from the three items.

### 2.6 Qualitative interviews

All included treatment participants were invited to participate in a qualitative interview. The interview included questions regarding practical aspects of the treatment, such as difficulties logging in to the treatment platform, and several questions regarding the acceptability of the intervention. The questions assessed each acceptability dimension described by Sekhon et al. (2017). In addition, four questions regarding cultural relevancy assessed this aspect of the treatment. These questions were based on the cultural relevance questionnaire (Salamanca-Sanabria et al., 2019) but were simplified to match the target group better. See Appendix B for the complete interview guide.

Regarding the non-treatment participants, the interviewee was observed during the login process to the platform by a research staff member to understand difficulties in the login process better. The interviewee read 2–3 modules and used the same questions as mentioned above regarding the acceptability and cultural relevancy of the treatment material.

The interviews of both the treatment and non-treatment participants were conducted by the first and last author, both male and licensed clinical psychologists, where one holds a PhD, and the other was a PhD candidate at the time when the interviews were conducted. Both interviewers had previous experience in conducting qualitative research. Both interviewers had extensive knowledge of the intervention and culturally adapted ICBT. One of the interviewers had been the therapist for the participants in the study, and the other interviewer had discussed the included participants in case management conferences. The participants were aware that both interviewers were a part of the research team for the study. The interviews were conducted either in Swedish, Dari or Farsi according to the participant's preference. Interviews with treatment participants were conducted over the phone. The interviews with the non-treatment participants were conducted in person, following the procedure described above. The interviews with the treatment participants were 27 min (range 7 to 46 min). The interviews with the non-treatment participants were 93 min (range 49 to 119 min). All interviews were recorded and transcribed. The transcriptions were not returned to participants for comment or corrections for data security reasons.

### 2.7 Statistical analyses

All descriptive and statistical analyses were done using SPSS v 27. A dependent *t*-test examined pre- to post- within-group differences. Given the exploratory nature of the current investigation, missing data were handled using complete case analysis.

### 2.8 Qualitative analysis

The qualitative data was analysed using thematic analysis (Braun and Clarke, 2006). The thematic analysis was conducted in a critical realist epistemological framework, focusing on semantic themes across the data set. The main question guiding the analysis was: how did the participants experience the acceptability and cultural appropriateness of the intervention? The first author coded all interview data, and the remaining authors then checked the coding. The codes were organised into themes by the first author and then reviewed by the entire research team in an iterative process, going back to the data material and the original codes to ensure that the analysis was representative of the data.

## 3 Results

### 3.1 Quantitative results

#### 3.1.1 Recruitment capability

Throughout the recruitment period, which spanned over five months, we could only include 15 participants.

#### 3.1.2 Attrition and adherence

Of the 15 participants who started the intervention, only 3 (20%) completed the post-treatment assessment. The three participants who completed the post-treatment assessment had completed 0, 2 and 4 modules, respectively. Moreover, adherence to the intervention was very low, with participants opening on average 1.7 modules and completing 0.9 modules with a range of 0 to 4 modules. Nine participants did not complete a single module, and two participants never logged in to the platform. Overall, this indicated the low feasibility of the intervention in its current form.

#### 3.1.3 Treatment effects

Due to the high attrition rate, it was not possible to analyse the effect of the intervention. Table 2 summarises study participant characteristics across the outcome measures used.

### 3.2 Qualitative results

**Fig. 2:**
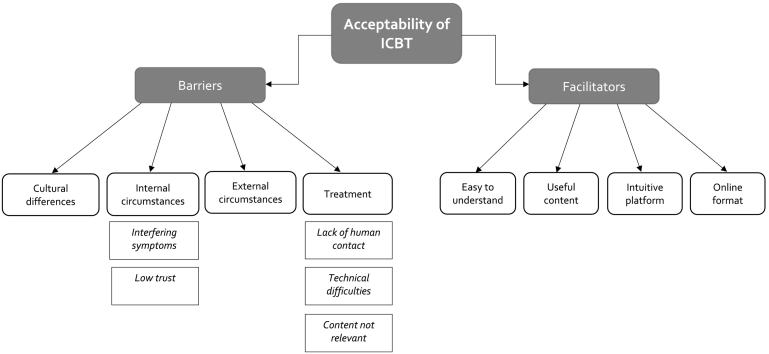
Structure of the thematic analysis.

The thematic analysis resulted in two main themes, *barriers* and *facilitators*, that each contained four themes and related subthemes, see Fig. 2. The four themes under *barriers* included *cultural differences*, *internal circumstances*, *external circumstances* and *treatment*, and the themes under *facilitators* included *easy to understand, helpful content*, *intuitive platform*, and *online format* see below.

### 3.3 Barriers

#### 3.3.1 Cultural differences

Several of the non-treatment participants expressed that there are cultural differences between the treatment program and the culture in Afghanistan, for example, regarding views of mental illness and differences regarding cultural practices around loss and mourning. One example of the latter mentioned by two participants was the Afghan custom of mourning for one year and not participating in any festive activities or listening to upbeat music during this period. However, while not necessarily deemed a barrier to participation, one of the participants also mentioned that cultural differences in gender roles could make some exercises challenging to perform for women.

“*[...] someone who is depressed needs to go out and be happy or do things she likes. But in our culture, a girl cannot do what she wants. The culture is a barrier. The problem is from the culture. [...] if one acts against the culture, the family and relatives hinder one.*”

(Non-treatment participant 2)

As the quotation illustrates, the conflict between the program and cultural norms related to gender roles could lead to conflict within families. The program targeted adolescents and young adults who are more dependent on the family than older participants. This theme was not found in any of the interviews of the treatment participants.

#### 3.3.2 Internal circumstances

The first subtheme, *interfering symptoms*, was found among two treatment participants and several non-treatment participants. Both groups of participants described that the text material required concentration and effort to understand and that symptoms of mental illness such as hopelessness, low energy, and lack of concentration and focus made it difficult to access the material. Two non-treatment participants also mentioned that the treatment material would be hard to use for people with severe mental health problems such as depression and was better suited for those with milder problems. The second subtheme, *low trust,* mentioned by one of the treatment participants and by several of the non-treatment participants, describes how both low trust in the helpfulness of text-based treatment, as well as in one's own ability to make use of the treatment, could act as a barrier to engaging with the treatment.

“[...] *why read a psychology text that can do good or not? Who wrote this? What does he or she stand for, why [did he/she] write this, maybe playing with my brain?*”

(Treatment participant 3)

As shown by this quote, this participant expressed distrust in the rationale behind the treatment and uncertainty regarding whether reading a psychological text could be of use, which in turn made him question the value of engaging with the treatment.

#### 3.3.3 External circumstances

Two of the treatment participants mentioned that external circumstances, such as school and work, with the resulting stress and lack of free time, acted as a barrier against spending more time on the treatment.

“*[...] nowadays I work quite a lot, I do not have [time] to do something like that [...] as soon as I wake up, yes, I do not have time to eat, but yes, I think I will [have time for the treatment] soon”.*

(Treatment participant 4)

This theme was not found in any of the interviews of the non-treatment participants.

#### 3.3.4 Treatment

The last theme under barriers contained three subthemes. The first subtheme, *lack of human contact*, was brought up by most of the treatment participants and the non-treatment participants. Overall, both groups of participants expressed a wish for more interaction with a therapist or support person of some sort, and three of the treatment participants mentioned that they had expected to have regular consultations with a therapist, either face-to-face or via telephone or video calls when signing up for the treatment. The value of human support was both in being listened to, which could give hope and energy, as a help to motivate one to engage with the written treatment material, and as an aid in understanding the treatment principles and receiving guidance.

“*You can read superficially and not understand, [if you] do not have the focus needed to understand. If you ask me more phone calls are needed [to understand the treatment] [...] It is much better talk live like we do now than to chat [...] I expected two to three phone calls a week to discuss my problems and get suggestions and guidance.*”

(Treatment participant 1)

As exemplified by this quotation, several participants expressed that phone conversations or video calls were superior to the text messaging system implemented in the treatment for the reasons given above. Moreover, the value of conversation was related to the lack of opportunities for these conversations in one's home country or during the period of flight before arriving in Sweden.

“*[...] I want to emphasise the conversation. I am thinking not only of myself but also of friends who have had depression or a friend who has recently lost their mother. They are looking for someone who listens to them. We [Afghans] who lived in Iranian society could not talk much. If you talked, you were told that it is silly, do not think of such things. There was no hope in the conversations, nothing to give you hope. [...] Having someone who listens to you and says you should fight and can get support, it might not help at first but hope makes you move.*”

(Treatment participant 3)

Consistent with this, two of the treatment participants also mentioned that the intake interview with the therapist had been a positive experience. The second subtheme, *technical difficulties*, was brought up by a majority of both treatment participants and non-treatment participants. The problems consisted mainly of difficulties setting up a password since it was not clear to participants that the password needed to contain both lowercase and uppercase letters and special characters to ensure password security. Finally, the third subtheme, *content not relevant*, was mentioned by two of the treatment participants and two non-treatment participants. One of the treatment participants expressed that while parts of the material were relevant for him, other parts were not.

“*Some things were not useful, did not fit with my personal problems. I didn't really understand. It was difficult and confusing. My problems differed from what was stated [in the text].*”

(Treatment participant 1)

As evident in the quote, this mismatch between the treatment content and his problems created confusion making it difficult to work with the treatment. The other treatment participant mentioned that one of the exercises did not make sense to him in his current life situation, while the two non-treatment participants found parts of the content a bit repetitive and some of the pre-treatment screening questions hard to understand.

### 3.4 Facilitators

#### 3.4.1 Easy to understand

The first theme was prevalent in most of the interviews of both the treatment participants and the non-treatment participants. Overall, participants expressed that the texts were easy to understand and written in a logical way that was easy to follow along, that the case examples were recognisable and pedagogic, and that reading in one's mother tongue helped with understanding the material.

“*It becomes more realistic when you have an example in the text [...] there was a girl who was an example, she was deciding on whether to meet her friend, and that you have to think about the consequences in the short term and in the long term [...] these kinds of examples are really good to have in the text*”.

(Non-treatment participant 3)

In addition, several participants mentioned that the poems included in the texts were culturally appropriate and added to the understanding of the material.

#### 3.4.2 Useful content

Most of the treatment participants and non-treatment participants mentioned that the text contained useful content. The exercises included in the modules were perceived as valuable and manageable by the non-treatment participants, while the treatment participants gave more general statements about the text containing valuable information and advice. One of the treatment participants and one of the non-treatment participants mentioned that reading the text had changed their way of thinking, as shown by the following quote.

“*[...] I learned a lot from the text and that, if I get this feeling again, I can easily handle [it] and think about what I read in the text, what it said and also recommend it to my friends.*”

(Non-treatment participant 1)

Thus, in this case, the new way of thinking also led to increased self-efficacy in managing difficult emotions.

#### 3.4.3 Intuitive platform

Apart from the difficulties logging in mentioned in a previous theme, most participants agreed that the treatment platform was easy to understand and navigate, with the only exception being one of the treatment participants who found it hard to navigate the menus using his mobile phone. However, this participant also stated that this was not a significant problem. One participant also mentioned that the messaging system was easy to use.

#### 3.4.4 Online format

Finally, in the last theme, two of the non-treatment participants mentioned various specific advantages of the online format, including the increased access for people living in remote locations where there is lack of on-site treatment resources and also the possibility to be anonymous, as shown by the following quote.

“*I think it's good to be able to write and send it away, it can be like this that some people find it a little difficult to talk about some things in person and so for some it's an advantage [...] some things are hard to talk about personally, especially for me or for us who have lived in another country, and we have so different cultures and so, some things that people talk about very openly in Sweden, we do not talk about in the same way as you do here.*”

(Non-treatment participant 3)

The above quotation also shows that the anonymity associated with the online format could be especially helpful in this particular target group, given the stigma surrounding mental health issues in Afghan culture. This theme was not found among the treatment participants.

## 4 Discussion

The present study is, to our knowledge, the first to investigate the feasibility and acceptability of an ICBT intervention targeting displaced Dari/Farsi-speaking adolescents and young adults. Compared with studies of ICBT in (Western) adult populations, the results suggest that such interventions may not be feasible and acceptable for this particular group, at least in their current form. Due to the high dropout rate (80%) and low compliance rates, it was impossible to analyse the treatment effects, including dose and response. In addition to the quantitative outcomes, qualitative interviews give insight into possible barriers and facilitators for implementing ICBT for this population, with the main barriers identified being a high mental health symptom burden and a wish for more human contact and support, possibly pointing to a need for a blended treatment format in order to increase adherence and acceptance of the intervention.

Regarding the feasibility of the intervention, we were only able to recruit 15 participants throughout the 5-month long recruitment period, despite trying to reach potential participants through various channels such as social media and various NGOs. There are, to our knowledge, no previous studies investigating attitudes towards digital health solutions among Dari and Farsi speaking youth, thereby making it hard to know if the results are indicative of a broader phenomenon or were more related to shortcomings in the recruitment strategy employed for the present study. Although the recruitment strategies employed in the present study have been successful in previous culturally adapted ICBT trials targeting adult populations (e.g. Lindegaard et al., 2020) and native Swedish youth populations (e.g. Berg et al., 2020a), different strategies may be needed to reach Dari and Farsi speaking refugee youth such as more targeted community outreach efforts (Chu and Leino, 2017), for example using social media influencers.

Moreover, the current feasibility study suffered from a high dropout rate (80%) and a low adherence rate, with participants completing on average 0.9 modules, indicating low feasibility and acceptance. These results are somewhat surprising given that a previous trial on a similar intervention targeting Arabic-speaking adults resettled in Sweden showed moderate to significant between-group effects on symptoms of depression and anxiety and with a substantially lower dropout rate of 39% (Lindegaard et al., 2020). Previous culturally adapted ICBT trials targeting adult non-western immigrant and refugee participants have overall demonstrated treatment effects, and adherence similar to standard ICBT interventions (Spanhel et al., 2021) and studies of ICBT targeting adolescent populations have produced treatment effects and adherence similar to those of adult populations (Berg et al., 2020b; Topooco et al., 2018). However, a critical difference in the present study was that 93% of participants were male, whereas previous studies on adolescents have had a large majority of female participants (Berg et al., 2020a; Topooco et al., 2018). While this rate reflects the gender distribution among unaccompanied Dari and Farsi speaking refugees overall, it is unclear how gender might interact with ICBT in adolescents and young adults with anxiety and depressive symptoms. However, this could be one part of the explanation for the low acceptance and feasibility found in the present study.

Another possible reason for the low acceptability of the intervention is that this had to do with insufficient cultural adaptation (Huey et al., 2014). However, this hypothesis was only partially supported by the findings in the qualitative study, as will be discussed below. It is also possible that psychosocial stress, such as the uncertainty regarding permanent residence permits currently faced by many Afghan youths (Swedish National Board of Health and Welfare, 2019), could interfere with participation in the trial. Qualitative findings support this, and it is also identified as a factor that can influence adherence and engagement with treatments in previous studies targeting migrants and refugees (Djelantik et al., 2020; Lindegaard et al., 2021). Finally, it is also possible that the education level of the participants acted as a barrier to engaging with the treatment in some way. Although most participants had completed Swedish upper secondary school, participants likely had varying educational opportunities before arriving in Sweden, which could make a text-based treatment less suitable for these participants.

The Thematic Analysis resulted in two main themes, barriers and facilitators that each containing four themes and related sub-themes. Overall, the two most prevalent and salient themes across the interviews regarding barriers were interfering symptoms, a subtheme of internal circumstances, and lack of human support, a subtheme of the theme treatment. Interfering symptoms concerned participants' experience of mental health symptoms such as hopelessness, low energy and lack of concentration constituting a barrier to reading and engaging with the text material that is a large part of the treatment. Instead, and connected to the other most prevalent theme, lack of human contact, most participants expressed a wish for more support and human interaction as a complement to the text-based treatment materials to be able to feel more hope, increase motivation to engage with the text, and aid in understanding and applying the treatment principles. Several participants also emphasised a wish for conversations, for example, via telephone or video calls and that being listened to was important for them and their peers. Similar themes regarding a wish for more human contact and interaction with a therapist have been found in previous qualitative studies of both adult and adolescent ICBT participants (Lenhard et al., 2016; Patel et al., 2020), and also regarding how initial motivations and sense of hope regarding the treatment seem to affect the experience of the treatment (Patel et al., 2020). However, these themes may be even more relevant for the present sample, possibly related to cultural variations in preference for oral or written communications (Yildiz, 2008).

Regarding explicit references to cultural differences, this was not clearly stated as a barrier in most cases except with regards to cultural norms regarding gender roles that could constitute a significant barrier for female participants. Also, the low trust in text-based psychological interventions brought forward by some participants could also be a sign of lacking cultural adaptation, indicating a need to provide more information on how this form of treatment can be helpful for the participants. However, overall, the results imply that the top-down cultural adaptation done before starting the intervention was successful in making the content of the material relevant and understandable.

The two most salient themes were named easy to understand and useful content regarding the facilitators. The first of these two themes, easy to understand, described how most participants found the text material easy to follow along, that the case examples aided in understanding the treatment principles and that reading in one's mother tongue stood preferable to reading in Swedish. In the second theme, useful content, most participants agreed that the texts contained valuable information relevant to common problems that many or their peers experienced. These findings align with previous ICBT studies for adolescent and adult participants, where many seem to find the treatment material relevant and informative (Bendelin et al., 2011; Berg et al., 2020a; Lenhard et al., 2016). Indeed, participants highlighted the value and relevance of the content for them, for instance, increasing self-efficacy (Richards et al., 2018).

Overall, relating to the acceptability model by Sekhon et al. (2017), the main problems regarding acceptability centred around the dimensions burden, opportunity cost and self-efficacy, since participants found the intervention overly demanding when suffering from mental health symptoms and that they required more support to from a therapist to make use of the treatment material. Also, the interview guide used for the present study (appendix B) can potentially be used for other researchers investigating the acceptability of ICBT for refugee youth populations.

### 4.1 Limitations

It is important to note that the small sample size and high dropout rate precluded the possibility of a quantitative evaluation of the efficacy of the treatment. However, the low recruitment capability and high dropout rate also constitute important information at this stage of the developmental process which will inform further development of this and similar interventions.

Similarly, the small sample for the qualitative interviews and the limited number of treatment participants who consented to participate in the interviews also make it difficult to know if the present analysis would be representative of the sample as a whole or if other important barriers and facilitators would be discovered with a larger sample of treatment participants. For this reason, we also decided to interview three non-treatment participants with similar demographics as the intended target population. However, the views expressed by these participants may differ from those in a more clinical group. At the same time, many of the barriers and facilitators named are familiar in the ICBT literature. It is also worth noting that only four of the fifteen participants were adolescents (<20 years), limiting the findings' generalizability to this age group.

Another limitation concerns the lack of a structured clinical diagnostic assessment of the participants. For this reason, it was not possible to assess what impact the diagnostic profiles of the participants had concerning the adherence and retention of the intervention or how this might have influenced the barriers and facilitators identified in the qualitative analysis.

A final limitation concerns the fact that we did not explicitly ask participants at post-treatment regarding potential adverse effects of the treatment. Given the pilot nature of the study, this would have contributed valuable information regarding the potential risks of this treatment approach when targeting refugee youth populations.

## 5 Conclusion and future research

To conclude, the present study highlights several important barriers and facilitators in the development of ICBT interventions for refugee youth. The difficulties recruiting participants and the low adherence in the present study indicated low feasibility and acceptance of the intervention in its current form. Qualitative interviews with the treatment participants and non-treatment participants with similar demographics as the treatment participants revealed that the treatment was culturally relevant and easy to understand. The most salient barriers across interviews were symptoms such as hopelessness and concentration difficulties interfering with engagement in treatment and a wish for more human support, for example, through telephone or video calls to complement the text-based treatment materials. Future developments of the intervention should investigate whether a blended treatment format with the inclusion of regular telephone or video calls with a therapist, or other on-site support such as a peer, can increase adherence to the intervention. Hopefully, having access to an increased therapist or peer support will lead to ICBT being a more acceptable and feasible intervention for this population.

## Declaration of competing interest

The authors declare that they have no known competing financial interests or personal relationships that could have appeared to influence the work reported in this paper.

## Appendix A Supplementary data

Supplementary material 1 Interview guide in English Image 1

Supplementary material 2 Top-down cultural adaptations Image 2
