# Supplementary material for: Investigating the potential of a novel internet-based cognitive behavioural intervention for Dari and Farsi speaking refugee youth: A feasibility study
Source: Internet Interv. 2022 Apr 1;28:100533. doi: 10.1016/j.invent.2022.100533 (PMC9006765; doi:10.1016/j.invent.2022.100533)
Supplement: Supplementary file 1 — Supplementary material 1 Interview guide in English [file mmc1.docx]

| Appendix A |  |  |
| --- | --- | --- |
| Top-down cultural adaptations made before pilot study |  |  |
| Module 1 - Introduction | Type of adaptation | Principle of adaptation |
|  | Added poem by Rumi in introduction | Conceptual equivalence |
|  | Added/changed examples of activities when exemplifying the ABC model and goal setting, for example change radio -> mobile phone. | Functional equivalence |
| Module 2 - Depression |  |  |
|  | Normalise feelings of sadness etc in the context of fleeing one’s home country. | Functional equivalence |
|  | Added/changed examples of behaviors associated with depression, for example staying home from school and not contacting friends | Functional equivalence |
|  | Added “Acceptance of negative thoughts and feelings” strategy, with rationale using Rumi “Guest house” poem. | Conceptual equivalence |
| Module 3 - Anxiety |  |  |
|  | Added poem by Hafez | Conceptual equivalence |
|  | Normalise feelings of anxiety when oneself or relatives are confronted with actual danger. | Functional equivalence |
|  | Add/changed examples of activities in exposure hierarchy, for example speaking up in class. | Functional equivalence |
|  | Change name of the case example. | Functional equivalence |
| Module 4 - Sleep problems |  |  |
|  | Normalise sleep problems in the context of difficult circumstances, such as being separated from one’s family. | Functional equivalence |
|  | Add/changed examples of activities that interfere with stimulus control, for example surfing on smartphone. | Functional equivalence |
|  | Change name of the case example. | Functional equivalence |
| Module 5 - Stress | Normalise stress in the context of difficult circumstances, such as being separated from one’s family. | Functional equivalence |
|  | Add/changed examples of activities that can cause stress, for example having uncertain residence status. | Functional equivalence |
|  | Clarify the difference between short-term and long-term stress with examples. | Functional equivalence |
|  | Add/change examples of recovery activities, and problematize the use of social media as a recovery strategy. | Functional equivalence |
|  | Change case example into younger person with school and migration related stress. | Functional equivalence |
| Module 6 - Worry and rumination |  |  |
|  | Normalise worry and rumination in the context of difficult circumstances, such as being separated from one’s family. | Functional equivalence |
|  | Change name of the case example. | Functional equivalence |
| Module 7 - Emotion regulation |  |  |
|  | Add examples that can lead to negative emotions, for example loss of a loved one, and the negative consequences of being unaware of emotions | Functional equivalence |
|  | Add examples of impulsive acting out, for example starting fights and addictive behaviors, and how this is related to lack of emotional awareness and avoidance. | Functional equivalence |
|  | Change name of the case example. | Functional equivalence |
|  | Added examples of acting contrary to the feeling, such as going to the dentist. | Functional equivalence |
| Module 8 - Traumatic memories |  |  |
|  | Change name and age of the case example. | Functional equivalence |
|  | Add examples of functional avoidance strategies when overwhelmed by emotions, for example watching movie. | Functional equivalence |
| Module 9 - Grief and separation anxiety |  |  |
|  | Added new module | Conceptual equivalence, Functional equivalence, Linguistic equivalence |
| Module 10 - Maintenence |  |  |
|  | Added case example | Functional equivalence |
|  | Added section on managing setbacks | Functional equivalence |
