# Supplementary material for: Investigating the potential of a novel internet-based cognitive behavioural intervention for Dari and Farsi speaking refugee youth: A feasibility study
Source: Internet Interv. 2022 Apr 1;28:100533. doi: 10.1016/j.invent.2022.100533 (PMC9006765; doi:10.1016/j.invent.2022.100533)
Supplement: Supplementary file 2 — Supplementary material 2 Top-down cultural adaptations [file mmc2.docx]

**Appendix B Interview guide in English**

**General questions (for treatment participants):**

Before I start, I wonder how you are affected by what is happening in Afghanistan (for those with an Afghan background)?

[if short of time] What was the reason that you discontinued the treatment? What can we do to improve?

How did you get information about the study?

How come you applied / registered? (search cause / problem searched for, motivation).

Which device do you use, mobile, computer, Ipad?

How was it for you to read on the home page? How easy was it to understand?

How was it for you to answer the questionnaires?

How was your experience of the telephone interview?

How was it for you to log in to the page for the SahaUng program for the first time?

How was the login the second time (SMS authentication)?

Which language did you choose?

What modules have you read?

How was it for you to understand the structure and orient yourself on the SahaUng webpage?

How was it for you to understand how to communicate with therapists (in the messaging system and by doing exercises and sending them)?

How should we set up the program / treatment for it to work for people like you?

**Questions for treatment participants and non-clinical participants:**

**Acceptability:**

What was your overall feeling / experience of the modules and the program? - Develop - give examples. AFFECTIVE ATTITUDE

How easy or difficult to understand did you experience that the texts were? - Is there anything that would make the texts easier to understand? INTERVENTION COHERENCE

How coherent did you feel the modules were? Was the message understandable? INTERVENTION COHERENCE

How strenuous did you experience it was to read the modules and take in the material? BURDEN, OPPORTUNITY COST

What obstacles did you experience in completing the treatment and reading the modules and when would you do the exercises each week? - What could make it easier for you? (Planning? Concentration? Confidence in one's own ability?) OPPORTUNITY COST, BURDEN, SELF-EFFICACY

To what extent has your needs been met? PERCEIVED EFFECTIVENESS

In a general, general sense, how satisfied are you with the help you have received? PERCEIVED EFFECTIVENESS

Do you have any other feedback you want to add that we have not asked about that you think would be good for us to know? - What would you like a treatment to look like in order to reach people like you?

**Cultural relevance:**

In different countries, cultures and life situations (eg being an asylum seeker) there are different ways of looking at and talking about mental illness: how well did you feel that the program matched your way of looking at mental illness and what you can do to feel better? ETHICALITY, CULTURAL RELEVANCE

To what extent could you recognize yourself in examples around how depression and anxiety can look in a person.

To what extent does the program contain relevant linguistic expressions, such as slang?

How relevant were the poems and metaphors used in the treatment?

To what extent was the material relevant based on their context and life situation?
